# High diversity and abundance of cultivable tetracycline-resistant bacteria in soil following pig manure application

**DOI:** 10.1038/s41598-018-20050-8

**Published:** 2018-01-24

**Authors:** Yijun Kang, Qing Li, Zhifeng Yin, Min Shen, Haitao Zhao, Yanchao Bai, Lijuan Mei, Jian Hu

**Affiliations:** 1 Environmental Science & Engineering, Yangzhou University, Yangzhou, Jiangsu P. R. China; 2 College of Marine and Bio-engineering, Yancheng Teachers University, Yancheng, Jiangsu P. R. China; 3 Jiangsu Key Laboratory for Solid Organic Waste Utilization, Nanjing Agricultural University, Nanjing, Jiangsu P. R. China

## Abstract

By performing a microcosm experiment mimicking fertilization, we assessed the dynamic distribution of tetracycline-resistant bacteria (TRB) and corresponding tetracycline resistance genes (TRGs) from pig manure (PM) to the fertilized soil, by culture-dependent methods and PCR detection. Cultivable TRB were most abundant in PM, followed by fertilized soil and unfertilized soil. By restriction fragment length polymorphism (RFLP) analysis, TRB were assigned to 29, 20, and 153 operational taxonomic units (OTUs) in PM, unfertilized soil, and fertilized soil, respectively. After identification, they were further grouped into 19, 12, and 62 species, showing an enhanced diversity of cultivable TRB in the soil following PM application. The proportions of potentially pathogenic TRB in fertilized soil decreased by 69.35% and 41.92% compared with PM and unfertilized soil. *Bacillus cereus* was likely widely distributed TRB under various environments, and *Rhodococcus erythropolis* and *Acinetobacter* sp. probably spread from PM to the soil via fertilization. Meanwhile, *tet*L was the most common efflux pump gene in both unfertilized and fertilized soils relative to PM; *tet*B(P) and *tet*36 were common in PM, whereas *tet*O was predominant in unfertilized and fertilized soil samples. Sequencing indicated that over 65% of randomly selected TRB in fertilized soil with acquired resistance derived from PM.

## Introduction

Due to broad-spectrum activities against a wide range of pathogenic bacteria in both humans and animals, tetracyclines (TCs) have been used in anti-infective therapy and breeding industry for many years^1^. TCs are more frequently used for treatment and prophylaxis, and even as growth inducers, in livestock than humans^2,3^, which results in the selection of resistant animal pathogens through horizontal gene transfer (HGT) by means of mobile genetic elements^4–6^. The average antibiotic consumption per Chinese is nearly 10 times that of American individuals, with markedly elevated consumption by pigs in China^7,8^. Consequently, animal manures possess the highest number of antibiotic resistance genes (ARGs), especially tetracycline resistance genes (TRGs)^9^. In rural China, pig manure is often applied as organic fertilizer directly to the soil without any treatment. As a major source of antibiotic pollution^8^, it leads to large-scale soil and water pollution, harming humans through the food chain^10–13^. Therefore, how to safely process pig manure before field application is of great interest in China. To achieve this, uncovering the transfer characteristics of ARGs from manure to the fertilized soil and analyzing the shift in hosts harboring TRGs are critical to understanding the vital factors affecting the biosafety of pig manure.

Multiple studies have assessed TRG distribution in various hosts by the metagenome sequencing technology. Zhu *et al*. found that *tet*Q, *tet*W, *tet*X, *tet*32, *tet*O, *tet*M, *tet*L, and *tet*G are most abundant in the soil^14^. Ghosh and LaPara demonstrated that the most common genes are *tet*L, *tet*A, *tet*M, and *tet*G^15^. Li *et al*. showed that *tet*M is central to the TRG network, and could be used as an indicator to quantitatively estimate the abundances of other TRGs^16^. In three populations, *tet*32, *tet*40, *tet*O, *tet*Q and *tet*W were found to be prevalent in all gut samples, with *tet*Q being the most abundant^9^. Jurado-Rabadán *et al*. revealed that *tet*M is the most common TRG in enterococci^17^. As for TRG hosts, different results were obtained by researchers. Gao *et al*. found that *Bacillus* is the most dominant genus in tetracycline-resistant bacteria (TRB) in aquaculture environment^18^. Huang *et al*. indicated that the majority of genera during anaerobic treatment of waste sludge are *Prevotella*, *Caldisericum*, *Pelobacter*, *Pseudomonas* and *Clostridium* with different pH levels^19^. These findings suggest that TRG distribution varies with samples, bacterial hosts, and environmental factors. However, the dynamic occurrence and distribution of TRGs and their hosts from pig manure to the fertilized soil remain unclear, although such knowledge would help understand the actual risk of TRG transmission from pig manure.

Metagenomics can provide information about the prevalence rates of species of interest, ARGs and mobile genetic elements in various environments, and help identify novel ARGs^20,21^. However, for accurate assessment of preferential ARG hosts and shift with environmental factors, the metagenomics approach seems to be unreliable, since high-throughput 16S rDNA sequencing cannot distinguish which DNA fragments come from ARB. This may lead to inaccurate associations of ARGs with their hosts. Meanwhile, using culture-dependent methods to uncover the dynamic distribution of ARGs from pig manure to the fertilized soil is feasible theoretically, although they are time-consuming. Besides, the traditional approach can probably provide information about bacterial hosts at the species level, with the possibility to further assess the evolutionary mechanism of ARGs at both the cell and gene levels.

In the present study, a microcosm experiment mimicking fertilization was performed to assess (i) the dynamic distribution of TRGs from pig manure to the fertilized soil and (ii) the preferential TRG hosts and shift during fertilization. The current findings may help elucidate the impact of pig manure on TRG distribution in the soil, also providing a basis for the further development of strategies to control TRGs.

## Materials and Methods

### Pig manure

Pig manure samples were collected from a pig farm with an eleven year feeding history in Qinfeng Town, Yangzhou City, which produces about 1,000 pigs yearly (pig products expanded since 2013). In normal feeds, TCs were added as production booster, and prophylactic or therapeutic agent, at a dose of 250 mg per kg feed. Daily feed consumption for each fattening pig was about 4% of body weight. Fresh pig manure excreted by adult male pigs was collected and transported to the laboratory for immediate use. By the HPLC-MS/MS method^22–24^, TC amounts in manure samples were 986.3 ± 39.4 μg kg^−1^.

### Microcosm experiment

Sterile Petri dishes (150 mm × 33 mm) containing 50-gram of pig manure, soil, and soil + pig manure, respectively (n = 3 per group), were prepared. Soil was collected from the upper 15 cm layer from barren land in Yangzhou University, with no fertilizer applied for over ten years. The characteristics of the soil samples were: pH 6.41; soil-water ratio, 1:1; organic matter, 11.04 g kg^−1^; cation exchange capacity, 8.96 cmol kg^−1^. After pulverization and sieving (2 mm), soil samples were mixed evenly with pig manure specimens in different treatments mentioned above, in Petri dishes at a rate of 0.4% according to the traditional fertilization recommendations. All three treatments were placed at 25 °C and incubated for 30 days, since most organic fertilizers exhibit fertilization efficiency within 15–30 days. The moisture content of each manure sample was adjusted to 55% using sterile ddH_2_O^25,26^. Moisture content was derived according to the following formula: water weight (g)/dry soil weight (g) × 100%, where dry soil weight was determined after drying to constant weight at 110 °C^27^.

### Counting, screening, and identification of TRB

Ten-gram samples (wet weight) were added to 90 mL of sterile dH_2_O, shaken at 120 rpm, and placed at room temperature for 20 min. The flask was left for 5 min to allow soil particles to settle, followed by a ten-fold serial dilution with sterile dH_2_O. A total of 100 µL of serial tenfold dilutions were plated on Luria-Bertani (LB)-TC agar medium, which comprised 1/10-strength LB^28^ agar supplemented with 16 μg ml^−1^ TC to grow cultivable TRB according to the Clinical and Laboratory Standards Institute (CLSI) document M100-S16^29^. Agar plates were incubated at 28 °C for 24 h, followed by routine counting. From plates with around 300 colonies each, individual colonies were picked, respectively, and streaked for single colony generation on LB-TC agar medium. Bacterial strains were separately stored at −80 °C in LB broth containing 20% glycerol.

Each pure culture was grown on a LB-TC agar plate for 12–48 h depending on growth rate; then, three loops of bacterial lawns were scraped into 200 μL of sterile ddH_2_O, followed by incubation in a water bath at 100 °C for 10 min and centrifugation at 8000 rpm for 3 min. The resulting supernatant was stored at −20 °C as DNA template. Nearly full length 16S rRNA was amplified with primers 27 f (5′-AGAGTTTGATCMTGGCTCAG-3′) and 1492r (5′-TACGGYTACCTTGTTACGACTT-3′)^30^. PCR was carried out in a 50 μL mixture system containing 10 μL DNA template, 0.2 mM of each dNTP, 0.4 μL of each primer, 1.25 U PrimeSTAR^®^ HS DNA Polymerase (TaKaRa, Dalian, China), and 1 × buffer (including Mg^2+^ at 1.5 mM final concentration). Amplification was performed on an Eppendorf Mastercycler (Perkin-Elmer, Inc., Waltham, MA) under the following conditions: initial denaturation at 94 °C for 5 min; 30 cycles of 94 °C for 1 min, 55 °C for 1 min, 72 °C for 1.5 min; final extension at 72 °C for 10 min. Amplification products were assessed by agarose gel electrophoresis (1% w/v agarose in Tris-Borate-EDTA buffer). The resulting PCR products were digested with the restriction enzyme *Hinf*I (TaKaRa, Dalian, China), separately, and distinguished according to different patterns mirrored by agarose gel electrophoresis at 1.2%. Only one randomly selected PCR product within the same *Hinf*I-digested fingerprint pattern was sequenced by Sangon Biotech. Co., Ltd., Shanghai, China. After comparison with the GenBank reference sequences, the obtained sequences for representative strains from different operational taxonomic units (OTUs) were deposited in GenBank using the submission tool Sequin. The accession numbers of TRB were KX981212 - KX981438, and KY048431 - KY048441 (duplicates were discarded, keeping only one representative strain per species). Phylogenetic trees were constructed using the neighbor-joining algorithm in MEGA5^31^.

To further identify each strain at the species level, strains within the same genus based on 16S rRNA gene sequences were respectively subjected to identification through their biochemical and morphological properties according to the Bergey’s Manual of Systematic Bacteriology.

### PCR detection of TRGs in TRB

PCR was employed to qualitatively assess currently known TRGs in TRB. Both genomic and plasmid DNAs were extracted with corresponding kits (Tiangen Biotech, Beijing) and mixed evenly. The mixed DNA was amplified for 44 target TRGs, including the 29 efflux pump genes *tet*A, *tet*B, *tet*C, *tet*D, *tet*E, *tet*G, *tet*H, *tet*J, *tet*K, *tet*L, *tet*V, *tet*Y, *tet*Z, *tet*A(P), *tet*30, *tet*31, *tet*33, *tet*35, *tet*38, *tet*39, *tet*40, *tet*41, *tet*42, *tet*45, *tet*AB(46), *tet*47, *tcr3*, *otr*B, and *otr*C; 11 ribosomal protection protein (RPP) coding genes *tet*M, *tet*O, *tet*Q, *tet*S, *tet*T, *tet*W, *tet*B(P), *tet*32, *tet*36, *tet*44, and *otr*A; 3 tetracycline-modifying enzyme genes *tet*X, *tet*34, and *tet*37; and *tet*U gene with unknown function. PCR amplification was performed in a 25 μl reaction system containing 2.5 μl of 10 × PCR buffer (including Mg^2+^ at a final concentration of 1.5 mM), 0.125 μl of each primer (30 μM) listed in Tables 1, 2 μl of DNA template, 0.25 μl of each deoxynucleoside triphosphate (80 mM), and 0.1 μl of *Taq* DNA polymerase (TaKaRa, Dalian, China). Amplification was performed on an Eppendorf Mastercycler (Perkin-Elmer Inc., Waltham, MA) under the following conditions: initial denaturation at 94 °C for 4 min; 35 cycles of 94 °C for 5 s, different annealing temperatures (listed in Table 1) for 45 s, 72 °C for 1 min; final extension at 72 °C for 6 min. Amplification products were separated by 1.5% agarose gel electrophoresis, stained with ethidium bromide, and visualized under UV light.

**Table 1:** PCR primers used in this study.

Primers	Targeted genes	Sequences (5′-3′)	Annealing temperature (°C)	Amplicon size (bp)	Reference
*tet*A-FW	*tet*A	GCGCGATCTGGTTCACTCG	61	164	^57^
*tet*A-RV		AGTCGACAGYRGCGCCGGC			
*tet*B-FW	*tet*B	TACGTGAATTTATTGCTTCGG	59	206	^58^
*tet*B-RV		ATACAGCATCCAAAGCGCAC			
*tet*C-FW	*tet*C	GCGGGATATCGTCCATTCCG	68	207	^59^
*tet*C-RV		GCGTAGAGGATCCACAGGACG			
*tet*D-FW	*tet*D	GGAATATCTCCCGGAAGCGG	68	187	^57^
*tet*D-RV		CACATTGGACAGTGCCAGCAG			
*tet*E-FW	*tet*E	GTTATTACGGGAGTTTGTTGG	61	199	^57^
*tet*E-RV		AATACAACACCCACACTACGC			
*tet*G-FW	*tet*G	GCAGAGCAGGTCGCTGG	65	134	^59^
*tet*G-RV		CCYGCAAGAGAAGCCAGAAG			
*tet*H-FW	*tet*H	CAGTGAAAATTCACTGGCAAC	61	185	^57^
*tet*H-RV		ATCCAAAGTGTGGTTGAGAAT			
*tet*J-FW	*tet*J	CGAAAACAGACTCGCCAATC	61	184	^57^
*tet*J-RV		TCCATAATGAGGTGGGGC			
*tet*K-FW	*tet*K	TCGATAGGAACAGCAGTA	55	169	^60^
*tet*K-RV		CAGCAGATCCTACTCCTT			
*tet*L-FW	*tet*L	TCGTTAGCGTGCTGTCATTC	55	267	^60^
*tet*L-RV		GTATCCCACCAATGTAGCCG			
*tet*V-FW	*tet*V	GCCTACGGTTTCATCCTGGC	65	351	^61^
*tet*V-RV		CGAGACCACCTTCGACAGCG			
*tet*Y-FW	*tet*Y	ATTTGTACCGGCAGAGCAAAC	68	181	^57^
*tet*Y-RV		GGCGCTGCCGCCATTATGC			
*tet*Z-FW	*tet*Z	CCTTCTCGACCAGGTCGG	61	204	^57^
*tet*Z-RV		ACCCACAGCGTGTCCGTC			
*tet*A(P)-FW	*tet*A(P)	CTTGGATTGCGGAAGAAGAG	55	676	^60^
*tet*A(P)-RV		ATATGCCCATTTAACCACGC			
*tet*30-FW	*tet*30	CATCTTGGTCGAGGTGACTGG	68	210	^57^
*tet*30-RV		ACGAGCACCCAGCCGAGC			
*tet*31-FW	*tet*31	CAATCACGCCCAAAAGAA	53	564	^62^
*tet*31-RV		TGTGCCATCCCAGTTTGT			
*tet*33-FW	*tet*33	ATGCGGTTCCGCTGAA	54	784	^63^
*tet*33-RV		GGAAAATGCGTCAGTGACAA			
*tet*35-FW	*tet*35	ATGCGCAAGACCGTCCTAC	54		^64^
*tet*35-RV		CACACACTAGTAACGGTCGAA			
*tet*38-FW	*tet*38	ATGAATGTTGAATATTCTAA	42	106	^65^
*tet*38-RV		TGGCTACAGAAATCAAT			
*tet*39-FW	*tet*39	CTCCTTCTCTATTGTGGCTA	47	701	^66^
*tet*39-RV		CACTAATACCTCTGGACATCA			
*tet*40-FW	*tet*40	CGGAGGAAGAGGACAAACCC	56	446	^67^
*tet*40-RV		TAAGCCGCTGCCGATAAGAC			
*tet*41-FW	*tet*41	AATGCGATCAATTTCCGCCG	55	166	This study
*tet*41-RV		CGGCGAACAGCAGATTAACG			
*tet*42-FW	*tet*42	TCTCGAGGATCACGAACCCT	55	128	This study
*tet*42-RV		ACTGGGACTCGATACACCCA			
*tet*45-FW	*tet*45	GCTGAGCCATCCACTCATTT	63	107	^68^
*tet*45-RV		TTTCCTCTTGAGCGTTTATGC			
*tet*AB(46)-FW	*tet*AB(46)	GCTTCTTGGACCTTGACGGA	55	580	This study
*tet*AB(46)-RV		GTTCCTGACTCATGGCCACA			
*tet*47-FW	*tet*47	GCGTTTGGCGTGGGTTTAAT	55	627	This study
*tet*47-RV		GACCCCTGTGGCATTGGTTA			
*tcr*3-FW	*tcr*3	CGCTCAGTTCGACAAGACCT	54	399	This study
*tcr*3-RV		GTCTCCATCGAGTTCGCCAT			
*otr*B-FW	*otr*B	CCGACATCTACGGGCGCAAGC	55	947	^69^
*otr*B-RV		GGTGATGACGGTCTGGGACAG			
*otr*C-FW	*otr*C	ATGAAGTTCCGCCGAATGNA	55	1860	^70^
*otr*C-RV		TCAGGTCTTCTTGCGGAACTT			
*tet*M-FW	*tet*M	ACAGAAAGCTTATTATATAAC	55	171	^59^
*tet*M-RV		TGGCGTGTCTATGATGTTCAC			
*tet*O-FW	*tet*O	ACGGARAGTTTATTGTATACC	60	171	^59^
*tet*O-RV		TGGCGTATCTATAATGTTGAC			
*tet*Q-FW	*tet*Q	AGAATCTGCTGTTTGCCAGTG	56	169	^59^
*tet*Q-RV		CGGAGTGTCAATGATATTGCA			
*tet*S-FW	*tet*S	GAAAGCTTACTATACAGTAGC	50	169	^59^
*tet*S-RV		AGGAGTATCTACAATATTTAC			
*tet*T-FW	*tet*T	AAGGTTTATTATATAAAAGTG	46	169	^71^
*tet*T-RV		AGGTGTATCTATGATATTTAC			
*tet*W-FW	*tet*W	GAGAGCCTGCTATATGCCAGC	64	168	^59^
*tet*W-RV		GGGCGTATCCACAATGTTAAC			
*tet*B(P)-FW	*tet*B(P)	AAAACTTATTATATTATAGTG	46	169	^59^
*tet*B(P)-RV		TGGAGTATCAATAATATTCAC			
*tet*32-FW	*tet*32	GAACCAGATGCTGCTCTT	57	620	^72^
*tet*32-RV		CATAGCCACGCCCACATGAT			
*tet*36-FW	*tet*36	TTTCTGGCAGAGGTAGAACG	57	250	^73^
*tet*36-RV		TTAATTCCTTGCCTTCAACG			
*tet*44-FW	*tet*44	AAAATAATCAACATTGGTATTCTTGCTCA	56	1927	^74^
*tet*44-RV		TAGTAACTTAATTTTCTTTTTTATTAAACATATGGCG			
*otr*A-FW	*otr*A	GAACACGTACTGACCGAGAAG	55	778	^69^
*otr*A-RV		CAGAAGTAGTTGTGCGTCCG			
*tet*X-FW	*tet*X	GAAAGAGACAACGACCGAGAG	56.5	131	^75^
*tet*X-RV		ACACCCATTGGTAAGGCTAAG			
*tet*34-FW	*tet*34	ATACGGGGATGCAAACTTCA	53	729	^63^
*tet*34-RV		ACGAGTGAGCTCTGATGTCTCTT			
*tet*37-FW	*tet*37	ATGGTTCGCTATTACTCTAAC	45	177	^76^
*tet*37-RV		ATCAGTCTCATATTTCGACA			
*tet*U-FW	*tet*U	ATGCAGCTAAGACGTGGC	54	317	^77^
*tet*U-RV		TTATTCGGTATCACTTCTCTGTC			

To confirm the TRGs base on size, five randomly selected bands for a particular TRG were excised from the agarose gel, followed by DNA recovery with a specific kit. The purified DNA was cloned into the pMD18-T vector (Takara Bio Inc.) and transformed into chemically competent *E. coli* DH5α. The extracted plasmid DNA from a positive clone was sequenced with universal primers by Sangon Biotech. Co., Ltd., Shanghai, China. After sequence comparison with BLAST, the confirmed PCR product was loaded on the gel as the positive control to verify other PCR products obtained for the same TRG.

### Correlation between the TRG percentage in cultivable TRB and TRG abundance obtained with the culture-independent approach

To assess if the culture-dependent method adopted in this study was reliable, the percentage of TRGs in TRB and TRG abundance obtained by real-time quantitative PCR (q-PCR) approach were assessed.

Total microbial DNA was extracted from manure, soil, and soil + pig manure samples with Power-Soil^TM^ DNA Isolation Kit (MO BIO Laboratories Inc., CA, USA) according to the manufacturer’s instructions. Six TRGs, namely *tet*B, *tet*C, *tet*M, *tet*O, *tet*T, and *tet*Z genes, were amplified with primers described in our previous work^23^. PCR was performed on a Bio-Rad Miniopticon (Bio-Rad Laboratories, CA, USA) with SYBR Green I for estimating the copy numbers of TRGs. A total of 20 μL reaction system containing 10 μL of iTaq Universal SYBR Green Supermix, 0.4 mM of each primer, and 10 ng of template DNA was set up. The amplification procedure consisted of 95 °C for 1 min, followed by 40 cycles of 94 °C for 10 s, 61 °C, 68 °C, 55 °C, 60 °C, 46 °C, and 61 °C for 45 s (corresponding to the *tet*B, *tet*C, *tet*M, *tet*O, *tet*T, and *tet*Z genes, respectively), and the subsequent disassociation curve generation. Data were analyzed for target genes from soil and/or manure samples as previously described^32^. Amplification efficiency (E) was estimated from the slope of the standard curve with the following formula: E = (10^−1/slope^) − 1^33^. PCR efficiency between 95% and 105% was adopted for further analysis^34^.

### Data analysis

Raw data were imported into Excel for analysis. Network visualization was performed on the interactive platform Cytoscape (version 3.2.0). Other graphs were obtained using Sigma Plot for Windows Version 10.0 (Systat Software, San Jose, CA, USA).

## Results

### Correlation between the percentage of TRGs in cultivable TRB and TRG abundance obtained by the q-PCR approach

**Figure 1:**
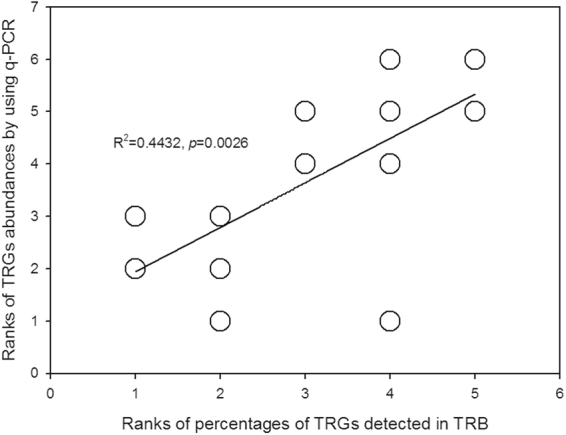
Correlation between the percentage of TRGs in cultivable TRB and TRG abundance obtained by the culture-independent approach. To avoid excessive differences in TRGs, the latter were ranked in each treatment.

It is necessary to assess whether the culture-based method adopted in this study is feasible. We therefore evaluated the correlation between the percentages of six randomly selected TRGs in cultivable TRB and their abundance levels obtained by the q-PCR approach. To avoid large differences in TRG abundance levels, the data obtained by the two methods were ranked and shown in Fig. 1. A good linear relationship was observed, indicating the reliability of the method used in this work.

### Cultivable TRB

Although cultivable TRB in pig manure were about four and three log units higher than those in unfertilized soil and soil + pig manure samples, OTU and species numbers were lower than those of soil + pig manure treatment (Table 2). Among the three treatments, the indices of cultivable TRB in the unfertilized soil were ranked lowest. These findings indicated that (1) relatively high abundance and low diversity of cultivable TRB were found in pig manure, and (2) cultivable TRB in the soil could be greatly enhanced by pig manure application.

**Table 2:** Cultivable TRB and species in the three samples.

Treatment	Cultivable TRB (lg cfu/g dry sample)^1^	OTUs numbers	Species numbers	Percentage of possible pathogen (%)
Pig manure	8.12^a^	29	19	47.37 (9/19)
Soil	3.98^c^	20	12	25.00 (3/12)
Soil + Pig manure	5.21^b^	153	62	14.52 (9/62)

OUT numbers were obtained by comparison of *Hinf*I-digested fingerprint patterns;

species numbers were obtained by 16S rRNA gene sequencing combined with biochemical and morphological properties.

^1^Means within columns followed by different letters are significantly different (Duncan’s test, *p* < 0.05).

**Figure 2:**
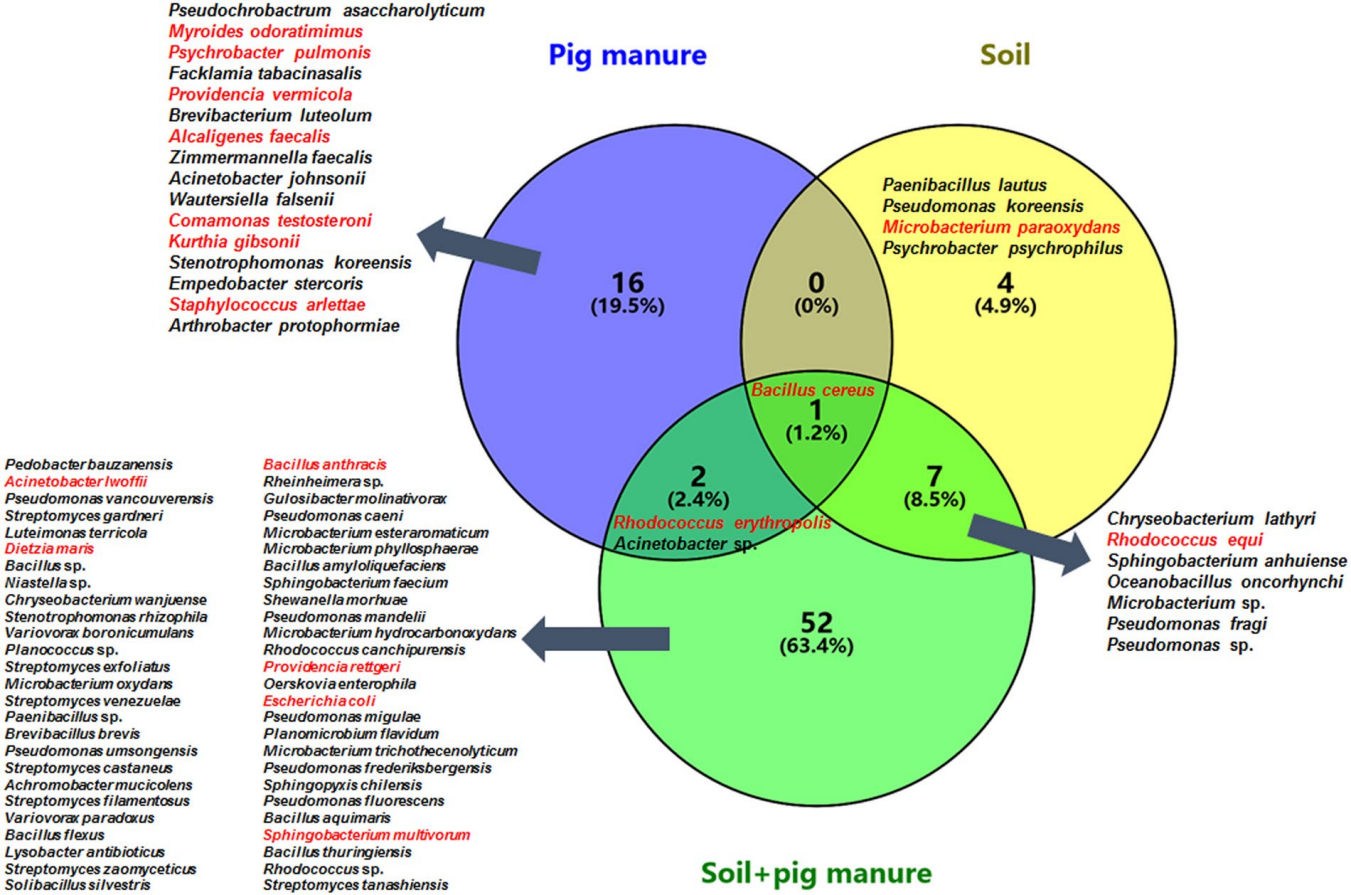
Venn diagram of shared TRB at the species level among the three samples. Species highlighted in red are potential pathogens.

The succession in cultivable TRB at the species level from pig manure to fertilized soil is shown in Fig. 2. Specific species in pig manure, soil, and soil + pig manure accounted for 19.5%, 4.9%, and 52% of all species, respectively. *Bacillus cereus* was present in all three samples, and represented relatively abundant TRB in the environment. Most species in pig manure were not present in the fertilized soil, which indicated that other factors such as nutrients played stimulatory roles in the enhancement of bacterial species. Seven species, including *Chryseobacterium lathyri*, *Rhodococcus equi*, *Microbacterium* sp., and *Pseudomonas fragi*, were found in both unfertilized and fertilized soils, suggesting that they may be stubborn soil species which are hard to control. *R. erythropolis* and *Acinetobacter* sp. were probably spread from pig manure to the soil via fertilization, and more attention should to be paid to these species.

### Frequency of the detected tetracycline resistance determinants

**Figure 3:**
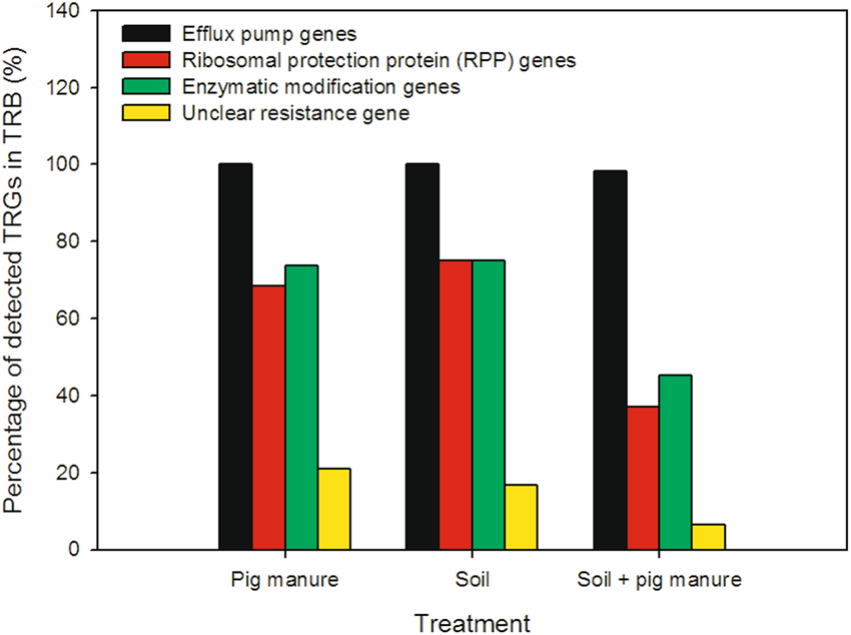
Percentages of the four TRG groups in TRB from the three samples.

In cultivable TRB derived from the three samples, except *tet*Y, *tet*38, *tet*45, *tet*44, and *tet*34, the remaining 39 TRGs were all found at different frequencies. In general, the detected species possessed efflux pump genes in all three samples, with most of them having multiple efflux pump genes (Fig. 3). For example, *Arthrobacter protophormiae* (accession number KY048441), *Stenotrophomonas koreensis* (accession number KY048438), and *Acinetobacter* sp. (accession number KY048432) had 13 such genes. The frequencies of RPP and enzymatic modification genes were similar in each sample, and these two TRG groups in the fertilized soil were about 50% lower than in pig manure and unfertilized soil samples. The TRG with unknown function (*tet*U) showed highest frequency in pig manure, followed by soil and fertilized soil samples.

**Figure 4:**
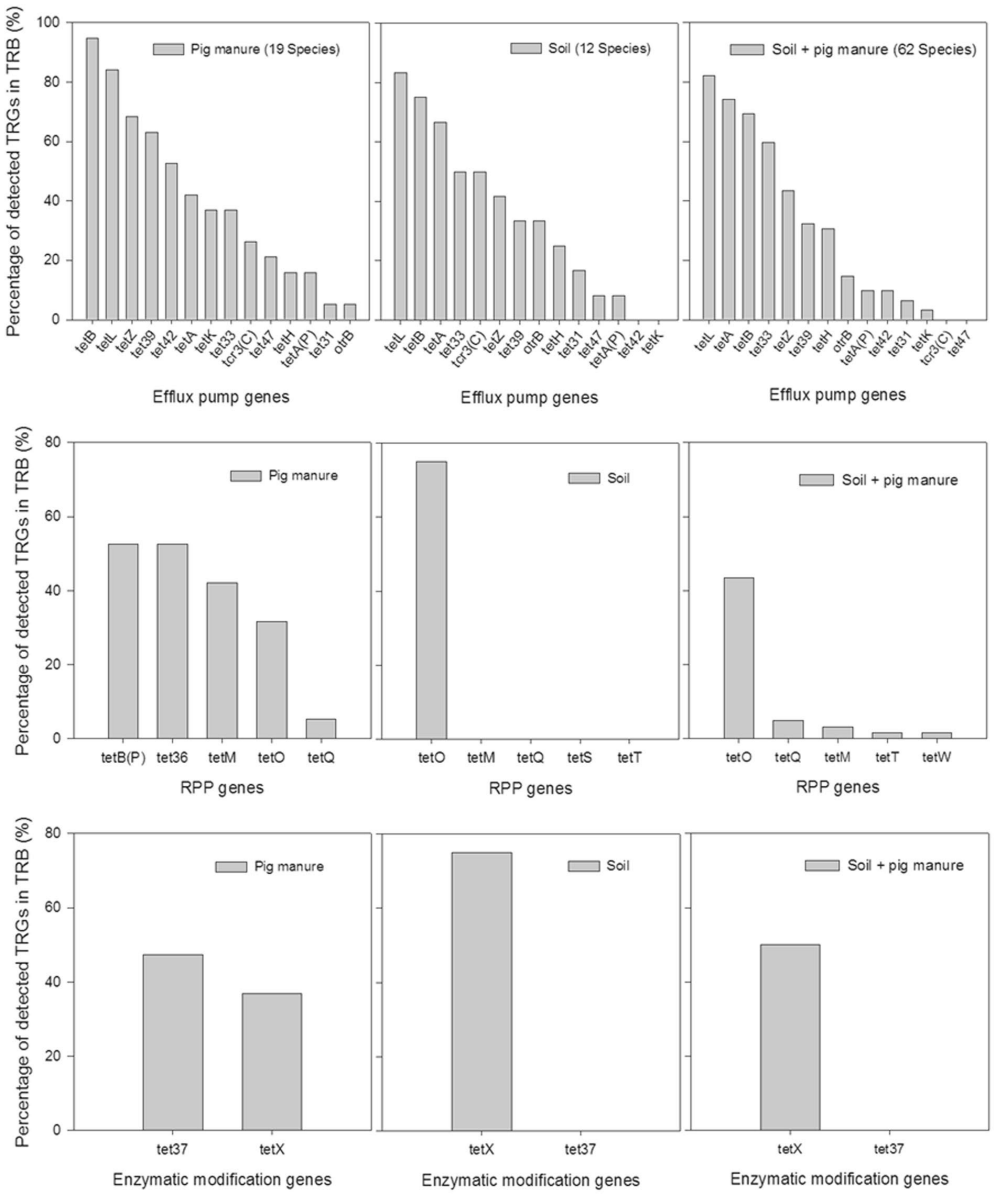
Percentages of each TRG in the three samples.

Of the efflux pump genes, *tet*B, *tet*L, and *tet*Z were the most common TRGs in pig manure, with frequencies 94.74%, 84.21%, and 68.42%, respectively; *tet*L (83.33%), *tet*B (75.00%), and *tet*A (66.67%) showed the highest frequencies in soil sample, and the top three efflux pump genes in the fertilized soil were *tet*L (82.26%), *tet*A (74.19%), and *tet*B (69.35%) (Fig. 4). As for RPP genes, *tet*B(P), *tet*36, *tet*M, and *tet*O were found at more than 30%, while in unfertilized and fertilized soil samples *tet*O absolutely had the highest frequency. Meanwhile, *tet*37 and *tet*X were both detected in pig manure at frequencies of 47.37% and 36.84%, respectively, while only the *tet*X gene was found in unfertilized and fertilized soil samples at frequencies 75.00% and 50.00%, respectively.

### Preferential hosts for different TRG groups

**Figure 5:**
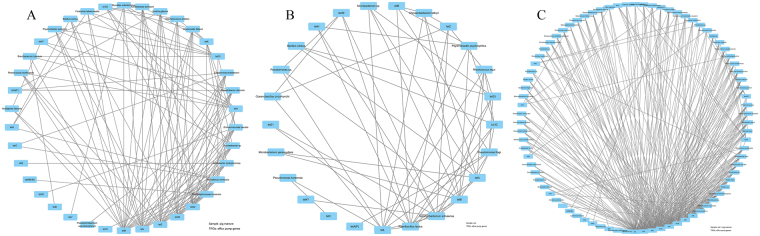
Network of efflux genes and their hosts isolated from pig manure (**A**), untreated soil (**B**), and soil + pig manure (**C**).

The networks of efflux pump genes and their hosts are shown in Fig. 5. The most complex network of TRGs and their hosts was obtained in the fertilized soil, followed by pig manure and normal soil. This finding indicated that pig manure application promoted the expression of efflux pump genes among diverse bacterial hosts. From pig manure and normal soil to fertilized soil, preferential hosts for efflux pump genes were changed from *Stenotrophomonas koreensis* (9 efflux pump genes), *Providencia vermicola* (9), *A. protophomiae* (8), *Acinetobacter* sp. (8), *Paenibacillus lautus* (10), *Sphingobacterium anhuiense* (10), *P. fragi* (7), and *Rhodococcus equi* (6) to *Variovorax paradoxus* (9), *Achromobacter mucicolens* (9), *Acinetobacter* sp. (8), *P. frederiksbergensis* (9), *Bacillus* sp., *et al*. (7). This finding suggested that *Acinetobacter* sp. was probably spread with fertilization, and changes in other preferential hosts for efflux pump genes in the fertilized soil might be stimulated by pig manure.

**Figure 6:**
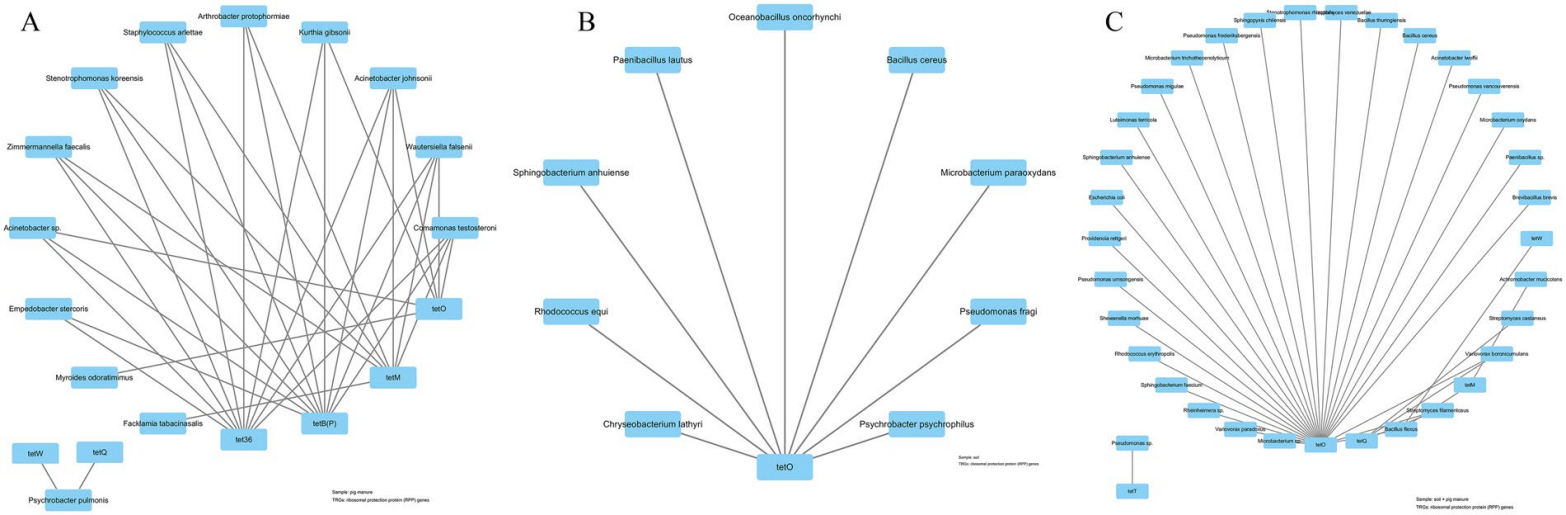
Network of ribosomal protection proteins (RPP) coding genes and their hosts isolated from pig manure (**A**), untreated soil (**B**), and soil + pig manure (**C**).

The preferential hosts for RPP genes were also changed obviously with pig manure application (Fig. 6). In unfertilized soil samples, all species had only *tet*O as RPP gene, while two groups of networks were distinguished in pig manure and fertilized soil specimens. In the fertilized soil, increases of *B. flexus*, *Streptomyces filamentosus*, *V. boronicumulans*, *S. castaneus*, *et al*. may be stimulated rather than introduced by pig manure.

**Figure 7:**
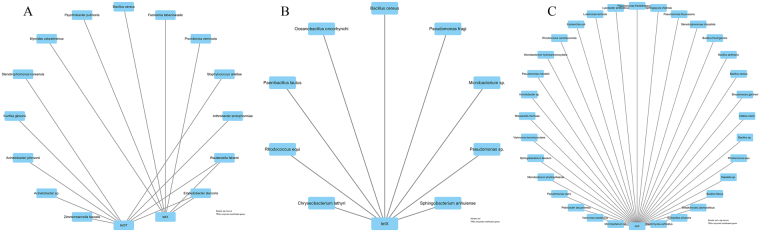
Network of tetracycline-modifying enzyme genes and their hosts isolated from pig manure (**A**), untreated soil (**B**), and soil + pig manure (**C**).

**Figure 8:**
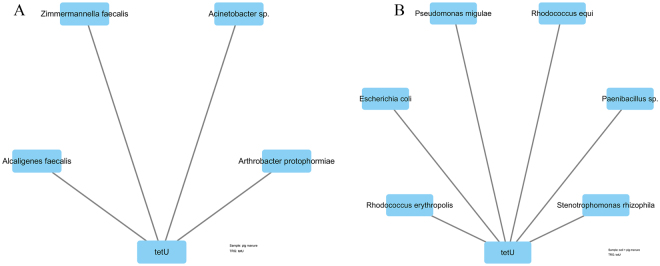
Network of a TRG with unknown function and its hosts isolated from pig manure (**A**) and soil + pig manure (**B**).

*Acinetobacter* sp. may also be the host for tetracycline-modifying enzyme genes introduced by pig manure (Fig. 7). *B. cereus* was a common host for genes in all three treatments. Except for *Microbacterium* sp. and *R. equi*, other species were possibly stimulated by pig manure. Besides, the hosts of the unknown TRG *tet*U seemed to be also induced by pig manure (Fig. 8).

## Discussion

### The negative effects of pig manure on TRG spread to the soil require special attention

Multiple studies have reported the high abundance and diversity of TRGs and/or TRB in pig manure and commercial organic fertilizers^14,22,35,36^. However, this study found that cultivable TRB in fertilized soil were three times more diverse than in pig manure and soil (Table 2), indicating that pig manure application does not only enhance TRG abundance but also, more importantly, could increase the diversity of cultivable TRB in the soil. This undoubtedly intensifies the negative effects of pig manure on the spread of TRGs to the soil. Using a metagenomics approach, Udikovic-Kolic *et al*. showed that manure-treated soil has less phylogenetic diversity of bacteria compared with NPK-treated soil^37^. Although bacterial diversity in fertilized and unfertilized soils was not assessed in the current study, it can be inferred that larger proportions of bacteria were tetracycline resistant in the fertilized soil compared with the untreated soil, with many of them harboring proto-resistance or silent resistance genes^38^, which change into the tetracycline-resistant type following manure application.

Intriguingly, pig manure application did not increase the percentage of pathogenic TRB in the soil (Table 2, Fig. 2); meanwhile, the diversity of pathogenic TRB decreased from 47.37% (pig manure) and 25.00% (untreated soil) to 14.52% (fertilized soil). On the one hand, some TRB with antagonistic effects became predominant following pig manure application, and may be capable of inhibiting sensitive pathogenic bacteria. For example, *B. amyloliquefaciens*^39^, *P. fluorescens*^40^, *B. thuringiensis*^41^, *S. tanashiensis*^42^, *P. vancouverensis*^43^, *Chryseobacterium wanjuense*^44^, *et al*. are known for such activities. In addition, most pathogenic TRB (except *B. cereus*), such as *Myroides odoratimimus* and *Alcaligenes faecalis*, in pig manure may be more adapted to the environment than to untreated or fertilized soil, since they are common in the gut environment^45,46^.

It can be inferred that *R. erythropolis* and *Acinetobacter* sp. were probably spread from pig manure to soil via fertilization, and more attention should be paid to these species. *R. erythropolis* can cause bloodstream infection^47^, and was firstly detected in pig manure. The high adaptability in distinct and even extreme environments of this TRB has been reported by many studies^48,49^; this may be the reason for its wide distribution. *Acinetobacter* sp. in this study was not accurately identified at the species level, but the relatively high amount of TRGs as well as the wide distribution traits in this species also requires attention. Besides, *B. cereus*, which possessed around 5 TRGs, was found in all three samples. *B. cereus* is an opportunistic pathogen capable of causing food poisoning^50^; however, it is often isolated for its potential to promote plant growth, and has been developed for commercial use^51,52^. Therefore, attention should be paid when using bio-agents containing this bacterium.

### TRGs have diverse and distinct hosts between pig manure and the fertilized soil, with a high risk of spreading TRGs via pig manure application

To date, little is known about the changes of TRGs from pig manure to the soil. As shown above, *tet*L was the most common efflux pump gene in both untreated and fertilized soils versus pig manure, which is partly consistent with Peng *et al*.^53^. Besides, *tet*42 and *tet*K were most common in pig manure followed by fertilized soil and untreated soil, suggesting that they could be introduced into the soil via fertilization. Differences of *tet*A and *tet*33 in the three samples were also obvious, indicating that the four genes *tet*42, *tet*K, *tet*A, and *tet*33 could be used as indicators for monitoring efflux pump genes in TRGs among various treatments. However, further investigation is required since (1) the above data were obtained by culture-dependent methods with possible predilection for TRB growth on specified media, and (2) bacterial cell numbers were not taken into account in this study.

For RPP genes, pig manure increased *tet*M and *tet*Q in the soil, in part corroborating our previous study using the PCR detection approach^23^. As shown above, *tet*B(P) and *tet*36 were common in pig manure but undetected in both untreated and fertilized soils, in disagreemnt with a previous study^53^. The *tet*36 gene was firstly indentified in swine manure pits^54^, and is seldom used as an indicator in soil environments, suggesting that it may be only common in pig manure, with reduced risk of spreading. A similar result was obtained for *tet*37, an enzymatic modification gene which is rarely found in TRB^55^. We firstly reported that *Zimmermannella faecalis*, *Acinetobacter* sp., *A. johnsonii*, *Wautersiella falsenii*, *et al*. isolated from pig manure had the latter TRG. Another such gene, *tet*X, was increased in soil after manure application, and may have potential roles in degrading TCs in soil environments. In addition, most TRB had more than one TRG in this study, and many of them may acquire mobile genetic elements (MGEs) for fitness in the presence of TCs in the fertilized soil. This process can, on the other hand, impose a metabolic burden on bacterial hosts^56^. With time, they may reduce in fitness because of growth delay in the fertilized soil with decreasing TCs; thus, discarding some MGEs could be a possible strategy to achieve recovery of the ecological niche, which can be a source of donor cells for TRGs. Sequencing of nearly full length *tet*L and *tet*X in radomly selected TRB (Tables 3 and 4) revealed that about 63.6% (7/11) and 66.7% (4/6) of hosts harboring *tet*L and *tet*X probably acquired them from other TRB, indicating a diversity of hosts as well as common spread events for TRGs among TRB. All *tet*L or *tet*X sequences derived from different hosts were highly homologous; in addition, the hosts were mostly found in pig manure or fertilized soil samples, indicating severe TRG diffusion and spread from pig manure to the soil via manuring. Overall, TRGs had diverse and distinct hosts in pig manure, untreated soil, and fertilized soil in this study, suggesting that the spread of TRGs from pig manure to the soil remains a public concern.

**Table 3:** GC-contents of genomic *tet*L in different hosts.

Host	GC-content of *tet*L	Genomic GC-content of TRB	Difference over 10%
*Bacillus thuringiensis* 8	35.3	35.0	No
*Oceanobacillus oncorhynchi* 32	34.7	39.3	Yes
*Rhodococcus erythropolis* 45	34.1	62.3	Yes
*Bacillus cereus* 91	34.0	35.2	No
*Bacillus aquimaris* 113	34.9	43.3	Yes
*Myroides odoratimimus* 122	34.4	34.1	No
*Psychrobacter pulmonis* 135	34.2	42.8	Yes
*Myroides odoratimimus* 140	33.9	34.1	No
*Rhodococcus canchipurensis* 199	34.0	65.3	Yes
*Alcaligenes faecalis* 229	33.8	56.7	Yes
*Stenotrophomonas koreensis* EMB15	33.9	66.1	Yes

*tet*L was amplified using the primer pair *tet*L-FW (5′-GTMGTTGCGCGCTATATTCC-3′) and *tet*L-RV (5′-GTGAAMGRWAGCCCACCTAA-3′).

**Table 4:** GC-contents of genomic *tet*X in different hosts.

Host	GC-content of *tet*X	Genomic GC-content of TRB	Difference over 10%
*Pseudomonas caeni* 14	38.3	48.3	Yes
*Pedobacter bauzanensis* 77	38.5	38.7	No
*Psychrobacter pulmonis* 135	38.3	42.8	Yes
*Lysobacter antibioticus* 152	38.3	67.0	Yes
*Facklamia tabacinasalis* 168	38.5	38.9	No
*Wautersiella falsenii* EMB5	38.7	32.1	Yes

*tet*X was amplified using the primer pair *tet*X-FW (5′-ATGACAATGCGAATAGATACAGACA-3′) and *tet*X-RV (5′-CAATTGCTGAAACGTAAAGTC-3′).

## References

[1] Chopra I, Roberts M (2001). Tetracycline antibiotics: mode of action, applications, molecular biology, and epidemiology of bacterial resistance. Microbiol. Mol. Biol. Rev..

[2] Sengeløv G (2003). Bacterial antibiotic resistance levels in Danish farmland as a result of treatment with pig manure slurry. Environ. Int..

[3] Thiele-Bruhn S (2003). Pharmaceutical antibiotic compounds in soils – a review. J. Plant Nutr. Soil Sci..

[4] Witte W (1998). Medical consequences of antibiotic use in agriculture. Science.

[5] Recchia GD, Hall RM (1995). Gene cassettes: a new class of mobile element. Microbiology.

[6] Roberts MC (1996). Tetracycline resistance determinants: mechanisms of action, regulation of expression, genetic mobility, and distribution. FEMS Microbiol. Rev..

[7] Li Y (2014). China’s misuse of antibiotics should be curbed. Br. Med. J..

[8] Larson C (2015). China’s lakes of pig manure spawn antibiotic resistance. Science.

[9] Hu Y (2013). Metagenome-wide analysis of antibiotic resistance genes in a large cohort of human gut microbiota. Nat. Commun..

[10] Lia C (2015). Occurrence of antibiotics in soils and manures from greenhouse vegetable production bases of Beijing, China and an associated risk assessment. Sci. Total Environ..

[11] Zhang H, Luo Y, Wu L, Huang Y, Christie P (2015). Residues and potential ecological risks of veterinary antibiotics in manures and composts associated with protected vegetable farming. Environ. Sci. Pollu. Res..

[12] An J, Chen H, Wei S, Gu J (2015). Antibiotic contamination in animal manure, soil, and sewage sludge in Shenyang, northeast China. Environ. Earth Sci..

[13] Hu X, Zhou Q, Luo Y (2010). Occurrence and source analysis of typical veterinary antibiotics in manure, soil, vegetables and groundwater from organic vegetable bases, northern China. Environ. Pollut..

[14] Zhu YG (2013). Diverse and abundant antibiotic resistance genes in Chinese swine farms. Proc. Natl. Acad. Sci. USA.

[15] Ghosh S, Ramsden SJ, LaPara TM (2009). The role of anaerobic digestion in controlling the release of tetracycline resistance genes and class 1 integrons from municipal wastewater treatment plants. Appl. Microbiol. Biotechnol..

[16] Li B (2015). Metagenomic and network analysis reveal wide distribution and co-occurrence of environmental antibiotic resistance genes. ISME J..

[17] Jurado-Rabadán S (2014). Detection and linkage to mobile genetic elements of tetracycline resistance gene tet(M) in *Escherichia coli* isolates from pigs. BMC Vet. Res..

[18] Gao P (2012). Occurrence of sulfonamide and tetracycline-resistant bacteria and resistance genes in aquaculture environment. Water Res..

[19] Huang H (2016). Distribution of tetracycline resistance genes in anaerobic treatment of waste sludge: The role of pH in regulating tetracycline resistant bacteria and horizontal gene transfer. Bioresour. Technol..

[20] Monier J-M (2011). Metagenomic exploration of antibiotic resistance in soil. Curr. Opin. Microbiol..

[21] Su JQ, Wei B, Xu CY, Qiao M, Zhu YG (2014). Functional metagenomic characterization of antibiotic resistance genes in agricultural soils from China. Environ. Int..

[22] Kang Y (2016). Impacts of supplementing chemical fertilizers with organic fertilizers manufactured using pig manure as a substrate on the spread of tetracycline resistance genes in soil. Ecotox. Environ. Safe..

[23] Kang Y, Gu X, Hao Y, Hu J (2016). Autoclave treatment of pig manure does not reduce the risk of transmission and transfer of tetracycline resistance genes in soil: successive determinations with soil column experiments. Environ. Sci. Pollu. Res..

[24] Andreu V, Vazquez-Roig P, Blasco C, Picó Y (2009). Determination of tetracycline residues in soil by pressurized liquid extraction and liquid chromatography tandem mass spectrometry. Anal. Bioanal. Chem..

[25] Tiquia SM, Tam NFY, Hodgkiss IJ (1996). Microbial activities during composting of spent pig-manure sawdust litter at different moisture contents. Bioresour. Technol..

[26] Liang C, Das KC, McClendon RW (2003). The influence of temperature and moisture contents regimes on the aerobic microbial activity of a biosolids composting blend. Bioresour. Technol..

[27] Cambardella CA (1994). Field-scale variability of soil properties in central Iowa soils. Soil Sci. Soc. Am. J..

[28] Silby MW, Winstanley C, Godfrey SA, Levy SB, Jackson RW (2011). *Pseudomonas* genomes: diverse and adaptable. FEMS Microbiol. Rev..

[29] Clinical and Laboratory Standards Institute. in *M100-S21* (Clinical and Laboratory Standards Institute, Wayne, PA, 2011).

[30] Weisburg WG, Barns SM, Pelletier DA, Lane DJ (1991). 16S ribosomal DNA amplification for phylogenetic study. J. Bacteriol..

[31] Tamura K (2011). MEGA5: Molecular evolutionary genetics analysis using maximum likelihood, evolutionary distance, and maximum parsimony methods. Mol. Biol. Evol..

[32] Nathani NM (2013). Comparative evaluation of rumen metagenome community using qPCR and MG-RAST. AMB Express.

[33] Bustin SA (2009). The MIQE guidelines: minimum information for publication of quantitative real-time PCR experiments. Clin. Chem..

[34] Yu Z, Yang J, Amalfitano S, Yu X, Liu L (2014). Effects of water stratification and mixing on microbial community structure in a subtropical deep reservoir. Sci. Rep..

[35] Munir M, Xagoraraki I (2011). Levels of antibiotic resistance genes in manure, biosolids, and fertilized soil. J. Environ. Qual..

[36] Guo F (2016). Impacts of human activities on distribution of sulfate-reducing prokaryotes and antibiotic resistance genes in marine coastal sediments of Hong Kong. FEMS Microb. Ecol..

[37] Udikovic-Kolic N, Wichmann F, Broderick NA, Handelsman J (2014). Bloom of resident antibiotic-resistant bacteria in soil following manure fertilization. Proc. Natl. Acad. Sci. USA.

[38] Perry JA, Westman EL, Wright GD (2014). The antibiotic resistome: what’s new?. Curr. Opin. Microbiol..

[39] Shao J, Xu Z, Zhang N, Shen Q, Zhang R (2015). Contribution of indole-3-acetic acid in the plant growth promotion by the rhizospheric strain *Bacillus amyloliquefaciens* SQR9. Biol. Fertil. Soils.

[40] Kurek E, Jaroszuk-Ściseł J (2003). Rye (Secale cereale) growth promotion by *Pseudomonas fluorescens* strains and their interactions with *Fusarium culmorum* under various soil conditions. Biol. Control.

[41] Raddadi N (2007). *Bacillus thuringiensis* beyond insect biocontrol: plant growth promotion and biosafety of polyvalent strains. Ann. Microbiol..

[42] Johnson LE, Dietz A (1968). Kalafungin, a new antibiotic produced by *Streptomyces tanashiensis* strain kala. Appl. Environ. Microbiol..

[43] Paul NC, Ji SH, Deng JX, Yu SH (2013). Assemblages of endophytic bacteria in chili pepper (*Capsicum annuum* L.) and their antifungal activity against phytopathogens *in vitro*. Plant Omics.

[44] Kim H-S (2012). Identification and characterization of *Chryseobacterium wanjuense* strain KJ9C8 as a biocontrol agent of Phytophthora blight of pepper. Crop Prot..

[45] Ravindran C, Varatharajan GR, Raju R, Vasudevan L, Anantha SR (2015). Infection and pathogenecity of *Myroides odoratimimus* (NIOCR-12) isolated from the gut of grey mullet (*Mugil cephalus* (Linnaeus, 1758)). Microb. Pathogenesis.

[46] Liu WT, Marsh TL, Cheng H, Forney LJ (1997). Characterization of microbial diversity by determining terminal restriction fragment length polymorphisms of genes encoding 16S rRNA. Appl. Environ. Microbiol..

[47] Baba H (2009). First case of bloodstream infection caused by *Rhodococcus erythropolis*. Appl. Environ. Microbiol..

[48] de Carvalho CCCR (2012). Adaptation of *Rhodococcus erythropolis* cells for growth and bioremediation under extreme conditions. Res. Microbiol..

[49] de Carvalho CCCR, Fatal V, Alves SS, da Fonseca MMR (2007). Adaptation of *Rhodococcus erythropolis* cells to high concentrations of toluene. Appl. Microbiol. Biotechnol..

[50] Hoffmaster AR (2004). Identification of anthrax toxin genes in a *Bacillus cereus* associated with an illness resembling inhalation anthrax. Proc. Natl. Acad. Sci. USA.

[51] Yánez-Mendizábal V (2012). Production of the postharvest biocontrol agent *Bacillus subtilis* CPA-8 using low cost commercial products and by-products. Biol. Control.

[52] Lalloo R, Moonsamy G, Ramchuran S, Görgens J, Gardiner N (2010). Competitive exclusion as a mode of action of a novel *Bacillus cereus* aquaculture biological agent. Lett. Appl. Microbiol..

[53] Peng S, Wang Y, Zhou B, Lin X (2015). Long-term application of fresh and composted manure increase tetracycline resistance in the arable soil of eastern China. Sci. Total Environ..

[54] Whittle G (2003). Identification of a new ribosomal protection type of tetracycline resistance gene, *tet*(36), from swine manure pits. Appl. Environ. Microbiol..

[55] Roberts MC (2011). Environmental macrolide–lincosamide–streptogramin and tetracycline resistant bacteria. Front. Microbiol..

[56] Heuer H, Schmitt H, Smalla K (2011). Antibiotic resistance gene spread due to manure application on agricultural fields. Curr. Opin. Microbiol..

[57] Aminov RI (2002). Development, validation, and application of PCR primers for detection of tetracycline efflux genes of gram-negative bacteria. Appl. Environ. Microbiol..

[58] Nemec A, Dolzani L, Brisse S, van den Broek P, Dijkshoorn L (2004). Diversity of aminoglycoside-resistance genes and their association with class 1 integrons among strains of pan-European Acinetobacter *baumannii* clones. J. Med. Microbiol..

[59] Aminov RI, Garrigues-Jeanjean N, Mackie RI (2001). Molecular ecology of tetracycline resistance: development and validation of primers for detection of tetracycline resistance genes encoding ribosomal protection proteins. Appl. Environ. Microbiol..

[60] Ng LK, Martin I, Alfa M, Mulvey M (2001). Multiplex PCR for the detection of tetracycline resistant genes. Mol. Cell Probes.

[61] Kyselková M (2012). Tetracycline resistance and presence of tetracycline resistance determinants *tet*(V) and tap in rapidly growing *Mycobacteria* from agricultural soils and clinical isolates. Microbes Environ..

[62] Bojesen AM, Bager RJ, Ifrah D, Aarestrup FM (2011). The rarely reported *tet*(31) tetracycline resistance determinant is common in *Gallibacterium anatis*. Vet. Microbiol..

[63] Agersø Y, Sandvang D (2005). Class 1 integrons and tetracycline resistance genes in *Alcaligenes*, *Arthrobacter*, and *Pseudomonas* spp. isolated from pigsties and manured soil. Appl. Environ. Microbiol..

[64] Miranda CD, Kehrenberg C, Ulep C, Schwarz S, Roberts MC (2003). Diversity of tetracycline resistance genes in bacteria from chilean salmon farms. Antimicrob. Agents Chemother..

[65] Truong-Bolduc QC (2015). Role of the t*et3*8 efflux pump in S*taphylococcus aureus* internalization and survival in epithelial cells. Infect. Immun.

[66] Agersø Y, Guardabassi L (2005). Identification of Tet 39, a novel class of tetracycline resistance determinant in *Acinetobacter* spp. of environmental and clinical origin. J. Antimicrob. Chemother..

[67] CHEN L (2013). Antimicrobial susceptibility, tetracycline and erythromycin resistance genes, and multilocus sequence typing of *Streptococcus suis* isolates from diseased pigs in China. J. Vet. Med. Sci..

[68] You Y, Hilpert M, Ward MJ (2013). Identification of Tet45, a tetracycline efflux pump, from a poultry-litter-exposed soil isolate and persistence of *tet*(45) in the soil. J. Antimicrob. Chemother..

[69] Nikolakopoulou TL (2005). PCR detection of oxytetracycline resistance genes *otr*(A) and *otr*(B) in tetracycline-resistant Streptomycete isolates from diverse habitats. Curr. Microbiol..

[70] Yu L (2012). Molecular cloning and functional characterization of an ATP-binding cassette transporter OtrC from *Streptomyces rimosus*. BMC Biotech..

[71] Wu N, Qiao M, Zhang B, Cheng W-D, Zhu Y-G (2010). Abundance and diversity of tetracycline resistance genes in soils adjacent to representative swine feedlots in China. Environ. Sci. Technol..

[72] Melville CM, Scott KP, Mercer DK, Flint HJ (2001). Novel tetracycline resistance gene, *tet*(32), in the *Clostridium*-related human colonic anaerobe K10 and its transmission *in vitro* to the rumen anaerobe *Butyrivibrio fibrisolvens*. Antimicrob. Agents Chemother..

[73] Eitel Z, Sóki J, Urbán E, Nagy E (2013). The prevalence of antibiotic resistance genes in *Bacteroides fragilis* group strains isolated in different European countries. Anaerobe.

[74] Abril C, Brodard I, Perreten V (2010). Two novel antibiotic resistance genes, *tet*(44) and ant(6)-Ib, are located within a transferable pathogenicity island in *Campylobacter fetus* subsp. *fetus▿*. Antimicrob. Agents Chemother..

[75] Speer BS, Shoemaker NB, Salyers AA (1992). Bacterial resistance to tetracycline: mechanisms, transfer, and clinical significance. Clin. Microbiol. Rev..

[76] Collins JR (2016). Periodontal pathogens and tetracycline resistance genes in subgingival biofilm of periodontally healthy and diseased Dominican adults. Clin. Oral Invest..

[77] Perreten V (2005). Microarray-based detection of 90 antibiotic resistance genes of gram-positive bacteria. J. Clin. Microbiol..

